# *In Vivo* Analysis of Inhibitory Synaptic Inputs and Rebounds in Deep Cerebellar Nuclear Neurons

**DOI:** 10.1371/journal.pone.0018822

**Published:** 2011-04-28

**Authors:** Fredrik Bengtsson, Carl-Fredrik Ekerot, Henrik Jörntell

**Affiliations:** 1 Section for Neurophysiology, Department of Experimental Medical Science, Lund University, Lund, Sweden; 2 NeuroNano Research Center, Department of Experimental Medical Science, Lund University, Lund, Sweden; The Research Center of Neurobiology-Neurophysiology of Marseille, France

## Abstract

Neuronal function depends on the properties of the synaptic inputs the neuron receive and on its intrinsic responsive properties. However, the conditions for synaptic integration and activation of intrinsic responses may to a large extent depend on the level of background synaptic input. In this respect, the deep cerebellar nuclear (DCN) neurons are of particular interest: they feature a massive background synaptic input and an intrinsic, postinhibitory rebound depolarization with profound effects on the synaptic integration. Using *in vivo* whole cell patch clamp recordings from DCN cells in the cat, we find that the background of Purkinje cell input provides a tonic inhibitory synaptic noise in the DCN cell. Under these conditions, individual Purkinje cells appear to have a near negligible influence on the DCN cell and clear-cut rebounds are difficult to induce. Peripheral input that drives the simple spike output of the afferent PCs to the DCN cell generates a relatively strong DCN cell inhibition, but do not induce rebounds. In contrast, synchronized climbing fiber activation, which leads to a synchronized input from a large number of Purkinje cells, can induce profound rebound responses. In light of what is known about climbing fiber activation under behaviour, the present findings suggest that DCN cell rebound responses may be an unusual event. Our results also suggest that cortical modulation of DCN cell output require a substantial co-modulation of a large proportion of the PCs that innervate the cell, which is a possible rationale for the existence of the cerebellar microcomplex.

## Introduction

For practical reasons, studies of synaptic integration and intrinsic neuronal properties have mostly been performed in excised pieces of brain tissue (brain slices/in vitro preparations). However, it has gradually become recognized that the information processing carried out by a neuron may change when it is provided with *in vivo*-like patterns of synaptic input [1], [2], [3]. So far, the main effects described have been an increase in the sensitivity and a decrease in the response times to a given input [1], [2], [3], [4], [5], [6]. However, the impact of synaptic noise on more profound intrinsic membrane responses remains to be elucidated.

One such intrinsic neuronal response is the postinhibitory, excitatory rebound response of the deep cerebellar nuclear (DCN) cells described *in vitro* [7], [8]. The rebound response is a dominating response *in vitro* and many theories of cerebellar function incorporate this response as a central feature of how the cerebellum works [9], [10], [11], [12].

However, in the cerebellum *in vivo* DCN cells receive input from 100's of Purkinje cells (PCs), which are spontaneously active at high rates, even in the absence of excitatory synaptic inputs [13]. This situation is likely to have a big impact on (1) how the DCN cells integrate synaptic input and (2) the conditions under which the rebound responses can be elicited. Although it is clear that rebound-like responses can be evoked *in vivo* with electrical stimulation within brain tissue [14], [15], the conditions and limits for evoking rebound responses *in vivo*, and thereby their potential functional roles under behaviour, have not been explored.

DCN cells in the anterior interposed nucleus (AIP) receive PC input from the C3 zone of the cerebellar cortex, for which the sagittal microzonal organization has been investigated in a particularly high level of detail [16], [17]. Each microzone receives climbing fiber input from specific cell groups in the rostral dorsal accessory part of the inferior olive (rDAO) [18]. The PCs of a specific microzone in turn have a specific innervation territory in the AIP [18], [19], [20], [21], [22], and the specificity in the olivo-cortico-nuclear connections forms the basis for the microcomplex organization of the cerebellar cicruitry [20], [22], [23], [24], [25]. In the present study, we take advantage of the detailed knowledge of the microcomplex organization for the rDAO-C3 zone-AIP system to obtain a controlled activation of the PC input to the DCN cells to characterize the limits and conditions for rebound activation. We also analyze the spontaneous and evoked IPSPs to explore the conditions for synaptic integration in DCN cells under a high background of PC inputs.

## Results

**Figure 1:**
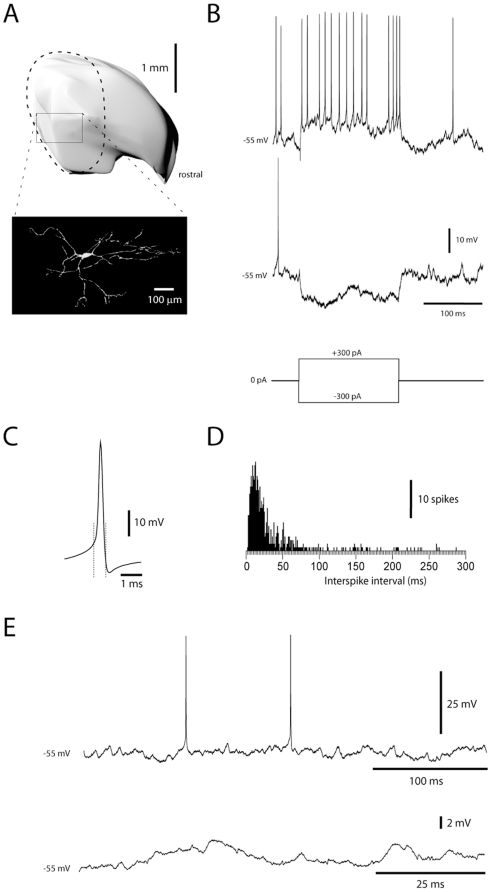
Identification of DCN neurons in whole cell recordings. (A) Location in the forelimb region (dashed line) of the anterior interposed nucleus (reconstructed in 3D) and morphological reconstruction of a DCN neuron, recorded with neurobiotin in the recording solution. (B) Responses of a DCN neuron to rectangular current step commands. (C) Average of 100 spikes recorded in one DCN neuron. Dashed lines indicate the start and the end of the spike, with the starting point defined as the point at which the second derivative of the voltage trace reached its peak value. (D) Frequency histogram of interspike intervals recorded in this cell. (E) Examples of spontaneous activity recorded at rest (0 pA bias current).

All DCN neurons in the present study were located in the forelimb area of the anterior interposed nucleus (AIP) in the decerebrated cat (Fig. 1A). We limited our study to neurons, which in intracellular recordings had intermediate spike-widths (0.61+/−0.07 ms, mean+/−sd.; N = 36) (Fig. 1C). In contrast, spike-widths of DCN neurons with broad spikes were 1.1+/−0.14 ms (N = 14) and for neurons with narrow spikes 0.49+/−0.06 ms (N = 3). Apart from spike widths, the neuron type we limited our analysis to also differed from cells with narrow and broad spikes by the distribution of their interspike intervals (21+/−12 ms, 46+/−11 and 104+/−51 ms, respectively) (Fig. 1C,D). DCN neurons with intermediate spike widths presumably correspond to the non-GABAergic projection neurons of the DCN [26].

Reconstructions of stained neurons (N = 3, Fig. 1A) were only partial because of difficulties in following the dendrites along their entire extent. Stained neurons were characterized by 7–9 dendrites that each could be followed for more than 0.4 mm and radiated in all directions. The membrane input resistance of the DCN neurons, measured from the voltage response obtained by current step commands (Fig. 1B), was 31+/−5.2 MOhm (N = 5). This is in the order of 1/10 to 1/20 of the values reported for rodents in the slice *in vitro* [27], [28]. The time-constant, measured from the voltage response to the hyperpolarizing current step, was 7.1+/−1.9 ms.

### Spontaneous IPSPs

**Figure 2:**
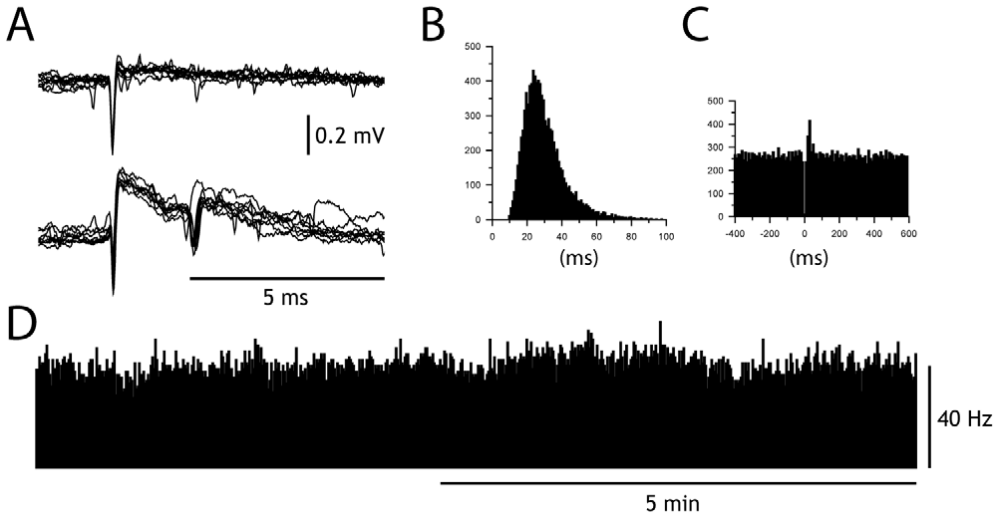
Activity in Purkinje cell projecting to the AIP. (A) Superimposed simple spike (top) and complex spikes from PC recorded in the C3 zone. (B) Frequency distribution histogram of interspike intervals for the simple spike. Bin width, 1 ms. (C) Peri-complex spike histogram of simple spike activity. Bin width 10 ms. (D) Histogram showing that the simple spike frequency remained relatively constant over time. Bin width, 1 s. We recorded the simple and complex spike activity of 16 PCs during more than 5 mins. The average firing frequency for simple spikes was 42+/−5.4 Hz and for complex spikes 2.8+/−0.5 Hz.

**Figure 3:**
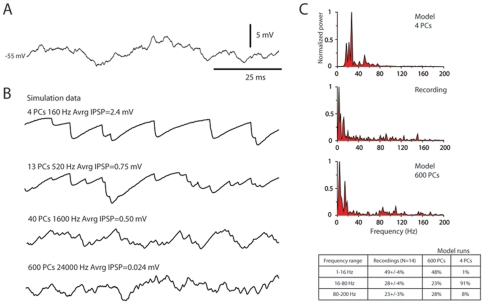
Non-synchronized, high frequency IPSP inputs generate smooth baseline. (A) Close-up of raw data. (B) Simulated data for varying number of PC inputs. The number of PCs, the average IPSP frequency and the average IPSP amplitude is indicated for each simulation run. (C) Power spectral density analysis for recorded signal and simulated signal with low and high number of PC inputs, respectively. Numerical comparisons were made for the relative power in different frequency intervals (inset table).

Purkinje cells (PCs) of the C3 zone in our preparation fire spontaneously at about 40 Hz (Fig. 2), similar to the PC firing rate observed in the awake animal at rest (see for example [29]). Given that a DCN cell in the cat receives at least in the order of 100's of PC inputs [23], [30], one would have expected the baseline to be heavily influenced by distinct IPSP events. However, this was not the case. On the contrary, the intracellular DCN recordings showed long periods with very low amplitude fluctuations, seemingly devoid of unitary synaptic inputs (Fig. 1E, 3A), which were only intermittently interrupted by spontaneous large IPSPs (cf. below). Periods of low amplitude fluctuations were defined as contiguous periods of 100 ms or more, during which the time derivative of the recorded membrane potential was free of deviations larger than two standard deviations. The root mean square (RMS) value of periods of low amplitude fluctuations was 1.4–1.7 mV, recorded in 14 different cells.

To investigate the conditions that could generate the periods of low amplitude fluctuations in the face of a high synaptic background activity we used a model of a DCN neuron, in which we simulated the integration of inhibitory synaptic inputs driven by recorded spontaneous spike times in PCs (Fig. 3). The simulation was carried out using different numbers of PCs (Fig. 3B). In order to calibrate the model against recorded data, we adjusted the synaptic weights of the afferent PCs so that the simulated membrane potential obtained a RMS value of about 1.5 mV, i.e. in the range of the recorded data. Notably, in order for the simulated membrane potential to stay within the same RMS range as the recorded signal, the mean unitary IPSP amplitude had to be very small, i.e. less than 0.1 mV in simulations with >200 PCs.

With this model, we found that periods of low amplitude fluctuations could only be obtained with a large number of non-synchronized PCs onto the DCN neuron (Fig. 3B, bottom). In contrast, if a low number of non-synchronized PCs converged on the DCN, the simulation indicated that the baseline would display a multitude of fast IPSP events (i.e. with deviations higher than 2 standard deviations, Fig. 3B, top). To further verify that the model produced an accurate reflection of the recorded low amplitude fluctuation periods in our DCN cells, we made a power spectral density analysis of the recorded and simulated subthreshold membrane potentials. Again, with a high number of PCs in the model, the power spectral density of the baseline activity became nearly identical to that of the DCN cell recording, which was not the case for simulations with a low number of PCs (Fig. 3C).

### Inferior olive stimulation

**Figure 4:**
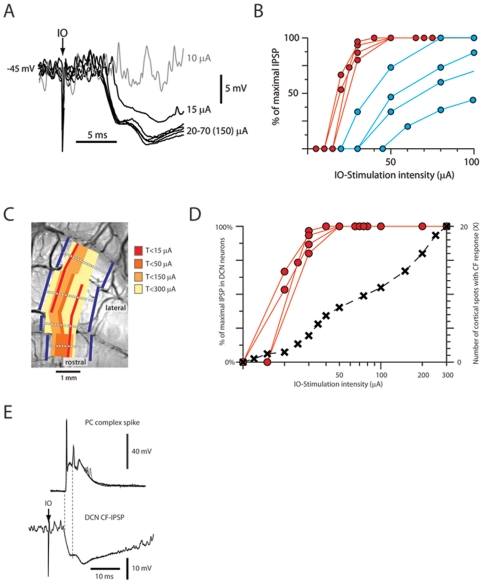
IPSPs evoked in DCN neurons by IO stimulation. (A) Superimposed averages of IPSP responses evoked at different intensities of IO-stimulation in the same DCN cell. Note that even at nearly 10 times the stimulus intensity for evoking a maximal response, the amplitude of the response did not change. The double-peaked nature of the evoked IPSP matched that of the complex spikes in the afferent PCs (see panel E). (B) Stimulus-response curves for DCN cells with low-threshold IO-evoked IPSPs (T<15 uA, red curves) compared with stimulus-response curves for IO-evoked IPSPs in DCN neurons with higher thresholds (blue curves). (C) Thresholds for cerebellar cortical climbing fiber field responses along the longitudinal axis of different folia of the forelimb area of the C3 zone. The recording sites are indicated on a photograph of the cerebellar anterior lobe sublobules Va-Vc, in which the forelimb area of the C3 zone is located. The borders of the C3 zone are indicated by thick blue lines. The yellow to red coloring indicates the specific thresholds for evoking climbing fiber responses according to the key at left. (D) Comparison between stimulus response curves for low threshold DCN cell IPSPs and the number of cortical spots with evoked climbing fiber responses (data averaged from 5 experiments, indicated by dashed line and crosses). (E) Maximal IO-evoked IPSPs consistently displayed two peaks, separated by about 3 ms (3.4+/−0.7 ms, N = 5, analyzed only for cells with identified, low thresholds for IO-evoked IPSPs). The timing of the two peaks of the IPSP paralleled the timing of the first two spikes recorded in the complex spikes from whole cell PC recordings (2.5+/−0.3 ms, N = 9).

In the next step, we wanted to test how synchronization of PC spike output was reflected in the membrane potential of the DCN cell. Because of the microcomplex organization of the cerebellum (see Introduction), in which the DCN cells only receive inputs from Purkinje cells within a single or a few sagittal microzones, electrical microstimulation within the inferior olive (IO) to simultaneously activate the PCs of single microzones should be an effective way to activate a large set of the inhibitory PC input to a DCN neuron. Using this approach, we found that thresholds for evoking DCN cell IPSPs, in the subset of DCN cells that were located within the same microcomplex as the stimulation electrode in the IO, were less than 15 uA. At current intensities of 50 uA or less we could evoke IPSPs of up to 20 mV in amplitude (Fig. 4A) (15+/−2.6 mV, N = 14). These IO-evoked giant IPSPs were maximal, since further increases in the IO stimulation intensity did not result in larger IPSPs (Fig. 4A,B). The DCN cells were recorded at a membrane potential of −45 to −50 mV to prevent the IPSPs from reaching the reversal potential for chloride. The reversal potential has been estimated to −78 mV in granule cells using the same recording solution (Jörntell and Ekerot, 2006), which is also close to the chloride reversal potential of −75 mV recorded for DCN cells *in vitro* with the perforated patch technique [31].

To verify that IO stimulation intensities of this magnitude only activated one or a few microzones of PCs in the cerebellar cortex, we recorded the thresholds for evoking climbing fiber field potentials across the entire mediolateral extent of the cerebellar C3 zone over several folia (Fig. 4C). With threshold stimulation, we could evoke climbing fiber field potentials along single cortical strips/microzones (i.e. sagittally connected bands of spots) in the medial and lateral parts of the C3 zone (c.f.([17], [32]). With increased stimulation intensities, the number of cortical microzones with activated climbing fiber input also increased, but at the point at which IO-evoked IPSPs were saturated, only 2–5 cortical microzones were activated (Fig. 4D, dotted/crossed line). As the stimulation intensity reached 300 uA, at least half of the microzones of the C3 zone was activated (Fig. 4D). Notably, in DCN cells for which the thresholds for IO-evoked IPSPs were higher (blue lines in Fig. 4B), saturation of the evoked IPSPs required considerably higher stimulation intensities.

### Spontaneous giant IPSP

**Figure 5:**
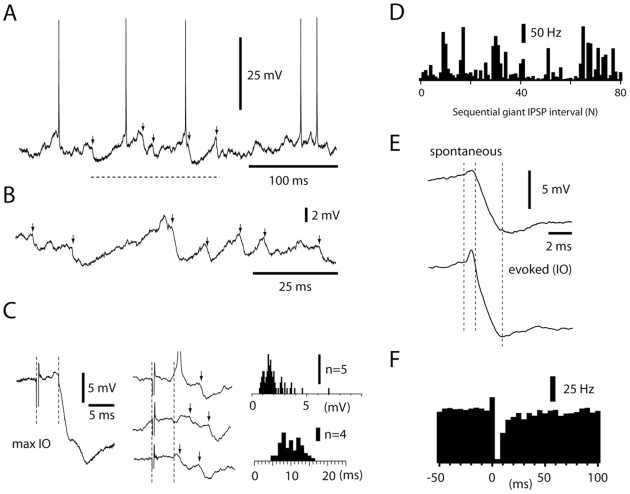
Properties of spontaneous giant IPSPs. (A) Raw trace illustrating a sample period (dashed line) during which spontaneous giant IPSPs (arrows) occurred. (B) Close-up of another period with spontaneous giant IPSPs. (C) Raw traces of IPSPs (arrows, middle panel) and histogram of IPSP amplitudes (right panel) evoked by threshold IO stimulation. For comparison, the averaged maximal IO evoked IPSP is illustrated to the left. Dashed lines indicate onset of stimulation and onset latency time of the maximal IPSP. Note the later, variable response latencies (summarized in histogram at bottom right) of the threshold IPSPs, which are likely due to that the direct neuronal activation is replaced with a more indirect, synaptic activation of the IO neurons as the stimulation intensity is reduced [65]. All recordings were made at −50 mV. Only IPSP events with an absolute amplitude >0.7 mV were analyzed. (D) Histogram of intervals between sequential giant IPSPs recorded during 3 minutes. The coefficient of variation of the intervals was 104%. (E) Average of the largest spontaneous giant IPSPs (N = 15) recorded from one DCN neuron and average of submaximal IO-evoked IPSPs (N = 10) recorded in the same neuron. Dashed lines indicate start of excitatory component, start of inhibitory component and peak of inhibitory component, respectively. (F) Peri-IPSP histogram of spike discharge relative to the onset of spontaneous giant IPSPs. Average from 9 DCN neurons. Bin width 5 ms.

The predominant low amplitude fluctuation periods of the DCN cell membrane potential trace (Fig. 3) were intermittently interrupted by episodes of large spontaneous IPSP-like events (Fig. 5A,B). These events could have very large peak amplitudes (typically 3–10 mV) when recorded at a membrane potential of −45 to −50 mV and are referred to as ‘spontaneous giant IPSPs’ below. Spontaneous giant IPSPs had a falling phase with a similar time course as submaximal IPSPs evoked by IO stimulation (Fig. 5C) (Since each olivary cell innervates on average 7 PCs [33], the IO evoked submaximal IPSPs must have been generated by at least 7 PCs, but possibly more since the IO stimulation could have activated more than one olivary cell). Spontaneous giant IPSPs typically occurred in bursts with highly irregular intervals (Fig. 5D) (the average CV for intervals between spontaneous giant IPSPs was 98%+/−6%) and at an average rate of 8–17 Hz (12.2+/−1.2, all data from 14 DCN recordings of at least 30 seconds each, 0 pA bias current). About 10% (9.8+/−6.1%) of the intervals between spontaneous giant IPSPs were longer than 1 s, excluding that they were generated by any single PC (Fig. 2). The high CV of the intervals and the occurrence of very long intervals suggested that the spontaneous giant IPSPs were generated by spontaneous discharges in the climbing fibers, which occur in similar patterns [34], [35]. The widely varying peak amplitudes further suggested that the spontaneous giant IPSPs were generated by synchronous activation of multiple olivary cells with a highly variable recruitment. Both spontaneous and IO-evoked giant IPSPs also shared the conspicuous feature of a precisely timed, preceding EPSP (peak amplitude: 0.5–1.5 mV (N = 10 cells)), which is elicited via the direct climbing fiber collateral input to the DCN cell [36]. The fact that the spontaneous giant IPSPs always were smaller than the maximal IO-evoked IPSPs suggested that they represented the activation of a subpopulation of the IO cells of the particular microcomplex to which the DCN cell belonged. The coactivation of small subpopulations of the IO cells could also explain how spontaneous giant IPSPs episodically could occur at much higher rates than the spontaneous climbing fiber discharge – bursts of giant IPSPs could represent the sequential activation of different subpopulations of IO cells innervating the microcomplex. Importantly, even though spontaneous giant IPSPs had a strong inhibitory effect on the spike activity, they failed to induce rebound excitation (Fig. 5F).

### Rebounds evoked by inferior olive stimulation

**Figure 6:**
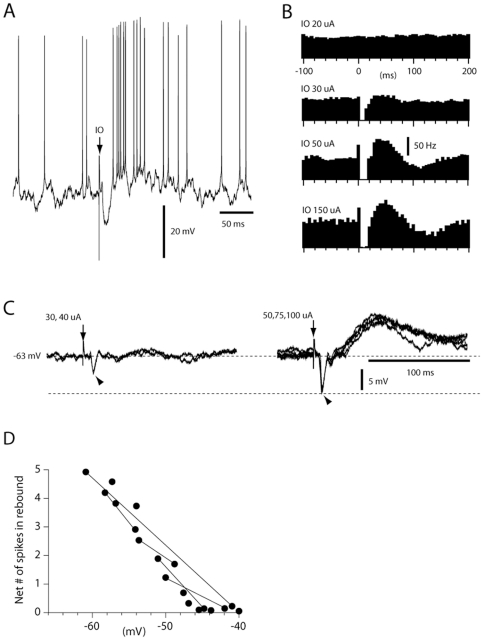
IO-evoked rebound responses. (A) Sample raw trace of rebound response evoked by maximal IO stimulation recorded at resting potential. (B) Spike responses evoked by subthreshold, submaximal, maximal and supramaximal IO stimulation (20, 30, 50 and 150 uA). Bin width, 5 ms. (C) Averages of responses evoked from the IO at different stimulation intensities recorded from another DCN neuron that was hyperpolarized to prevent spiking, Note all-or-nothing character of the rebound response as the IPSP (arrowheads) became maximal. (D) Relationship between membrane potential and the magnitude of the rebound response (expressed as net number of spikes, beyond prestimulus baseline, per stimulation). Lines indicate data obtained from the same cell at different membrane potentials.

In contrast, in cells in which IO stimulation evoked saturated IPSPs (Fig. 4), rebound responses were always evoked (Fig. 6) (the spike inhibition was 88%+/−7.8% for 10–15 ms, followed by a rebound of +72+/−14% lasting at least than 10 ms, N = 14). This response was hence similar to the rebound depolarizing response that has been described *in vitro* [7], [8] in that it appeared to require a preceding hyperpolarizing response beyond a threshold magnitude (Fig. 6B,C). To directly investigate whether the rebound responses were generated by the intrinsic membrane properties of the DCN cells, we compared the responses evoked at different membrane potentials (Fig. 6D). If the rebound response was directly driven by excitatory synaptic input, a hyperpolarized membrane potential would be expected to result in a decrease in the spike response after the IO stimulation. In contrast, we could systematically observe a clear INCREASE in the rebound spike response when the cell was hyperpolarized. Conversely, when the cells were depolarized there was a clear reduction of the rebound response (Fig. 6D). A possible interpretation of these findings is that hyperpolarization leads to a higher degree of deinactivation of low threshold calcium channels, which in turn could lead to a boost of the rebound [37].

Since the magnitude of IPSPs in whole cell recordings could potentially be abnormal due to the uncertainty of the estimates of the normal intracellular chloride concentrations *in vivo*, we also made a large number of extracellular metal electrode recordings (N = 174). In neurons with low-threshold inhibitory responses evoked from the IO (N = 36), maximal inhibitory responses (net change in spike activity −87%+/−3.7% during 13+/−1.5 ms, mean+/−sem) were evoked by an increase of the stimulation intensity of only 0–20 uA from the threshold, i.e. there was a similar stimulus-response relationship as in the intracellular records (Fig. 6A,B). In addition, in all these cases (N = 36), maximal IO-evoked inhibition was associated with prominent postinhibitory excitatory spike responses (net change in spike output +124%+/−22% during 26+/−8.2 ms). At the point at which IO stimulation evoked a maximal inhibitory response the rebound response was saturated, i.e. it did not increase when the stimulation intensity was increased by 2–3 times (Fig. 6B). In all cases where the inhibitory response evoked from the IO was graded (N = 11), we observed that submaximal inhibition, even though it provided strong spike suppression (−67%+/−5.7%), failed to trigger a rebound response (i.e. no increase in post-inhibitory spike activity beyond 2 SDs of prestimulus activity) (Fig. 6B).

**Figure 7:**
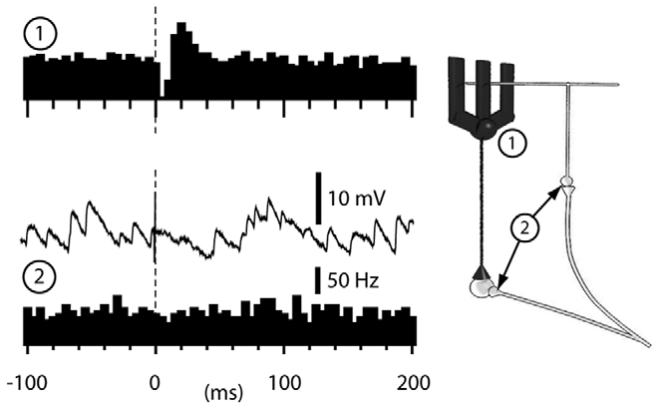
IO-evoked responses in cortical neurons. Simple spike responses of a PC (1) and MF-EPSP responses of a granule cell recorded in the whole cell mode (2) to IO stimulation. The granule cell data is shown both as a sample raw trace (recorded at −65 mV) and as a peristimulus histogram of EPSPs with IO-stimulation at 150 uA, which evoked substantial local climbing fiber field potentials in the cortex. Inset illustrates that the mossy fibers that innervate the DCN cells are collaterals of the mossy fibers that innervate the granule cells (2). Bin widths in all histograms, 5 ms.

IO-evoked rebound excitatory responses could not be explained by the discharge patterns of PC simple spikes or mossy fibers (Fig. 7). IO stimulation evoked a weak post-complex spike facilitation of simple spike output (the facilitation was +43%+/−6% (mean+/−sem, N = 14 cells) so the amount of PC inhibition would actually increase at the time of the DCN cell rebound response. Similarly, the mossy fiber EPSP responses in whole cell recordings from 72 granule cells in the C3 zone showed no post-IO-stimulation change (at one standard deviation vs. prestimulus activity; Fig. 7, bottom).

### DCN cell responses to electrical stimulation in the climbing fiber receptive field

**Figure 8:**
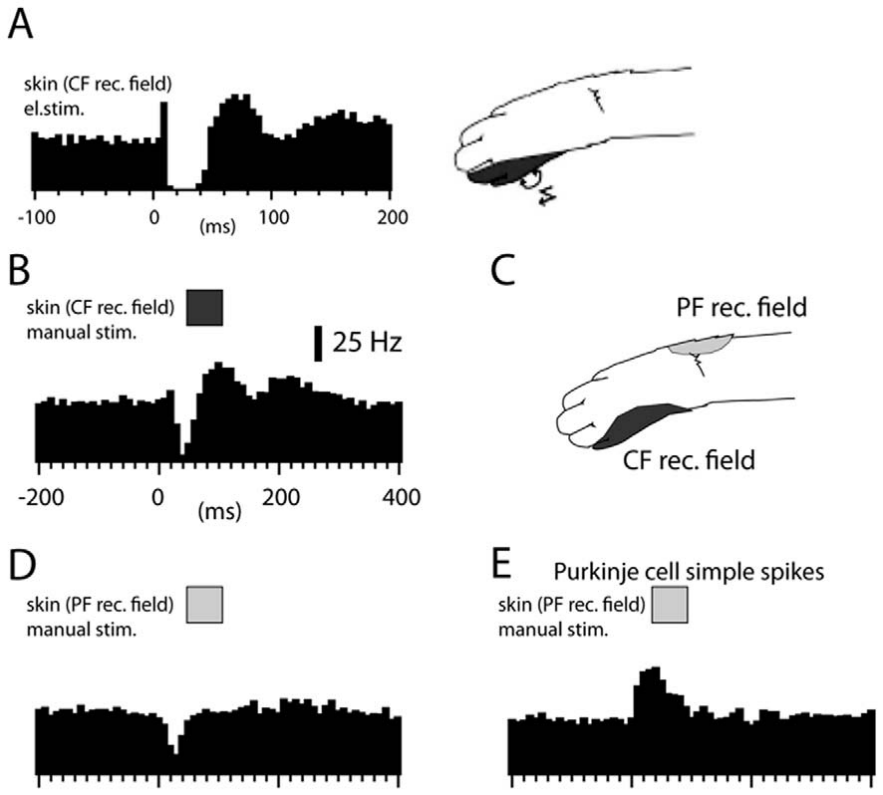
DCN cell responses to skin stimulation. (A) Spike responses of a DCN cell to electrical skin stimulation (single shock, 0.2 ms) within the climbing fiber receptive field. (B) Responses evoked by manual skin stimulation (50 ms) in the climbing fiber receptive field. (C) Locations of the parallel fiber and climbing fiber receptive fields for the PC input to the DCN neuron. (D) Response evoked by manual skin stimulation in the PC-parallel fiber receptive field. (E) For comparison, a peristimulus histogram of PC simple spike responses to manual skin stimulation within the parallel fiber receptive field. Histogram bin widths, 5 ms in (A), otherwise 10 ms.

The Purkinje cells of an entire microzone/microcomplex can also be activated by using skin stimulation in the specific receptive field of the climbing fibers of that microzone [17], [19]. A particularly effective way to evoke synchronized climbing fiber input is to use electrical stimulation of this receptive field [19]. As shown in Fig. 8A, this approach can be used to evoke a spike response in the DCN cell that is very similar to that evoked from the IO (Fig. 6B), i.e. initial excitation followed by strong inhibition and then a postinhibitory rebound. The climbing fiber receptive field of the DCN cell was defined as the skin site from which this type of response (spike suppression of 100% for at least 20 ms followed by a rebound of at least +50% for 30 ms, analyzed for 16 cells) could be evoked using electrical skin stimulation.

### DCN cell responses to manual activation from specific skin areas

We also investigated the responses of DCN cells to more natural patterns of synaptic input evoked by manual skin stimulation. Manual skin stimulation within the climbing fiber receptive field evoked the same response sequence (a spike activity depression of −84+/−3.6% followed by a postinhibitory rebound of +46+/−5.5%, lasting for 22+/−4 ms and 53+/−8 ms, respectively (N = 16)) as IO-stimulation (Fig. 8B). In the PCs of the C3 zone, also the parallel fiber (PF) input is evoked from a small receptive field. Importantly, the location of the PF receptive field is specific to (and also ‘opposite’ to) that of the climbing fiber receptive field of the PC (Fig. 8C), meaning that the PCs of a microzone can also be driven by PF input from one specific skin area [38], [39], [40]. However, in contrast to the responses evoked from the climbing fiber receptive field, manual skin stimulation of the PF receptive field of the PC input evoked inhibition (as expected) but not rebound. The spike reduction evoked from this skin area was −54%+/−8.8% (lasting for 46+/−3.0 ms) but the postinhibitory activity was unchanged (1.3%+/−0.7%, measured over the 50 ms that immediately followed the inhibition) (N = 16) (Fig. 8D).

## Discussion

In the present study, we used *in vivo* whole cell patch clamp recordings from presumed excitatory projection cells of the anterior interposed nucleus to explore the integration of PC inhibitory synaptic inputs and the conditions for evoking rebounds. We found a number of indications that individual PC IPSPs *in vivo* are exceptionally small. At least during rest, the PC inhibitory inputs seemed to be provided in a relatively tonic fashion with a low degree of synchronization between the different PCs (in accordance with observations on PC simple spike firing *in vivo* [41], [42]). Synchronized PC input, which was effectively achieved with inferior olivary stimulation, resulted in very large compound IPSPs in all of our DCN cells. Strong rebound responses required near maximal IO-evoked IPSPs, most likely involving synchronous activation of all or most PC inputs onto the DCN cell.

### PC-to-DCN cell convergence

A critical factor in the understanding of DCN cell synaptic integration is the number of convergent PCs onto each cell [30]. estimated the number of PC synapses on an interpositus neuron in the cat to 18,000, but used only indirect numerical estimates. A detailed electron microscopy study in the mouse [43] found that the number of GABA boutons per um^2^ is 0.023. Since the somatic diameter of stained DCN cells (this study and [44]) is about 35–40 um, the surface area of the soma is about 15000 um2. On the cell body alone, there should hence be more than 200 PC synapses. Each DCN cell has about 8 dendrites (Fig. 1, see also [44]) that each is at least 500 um long. About 70% of the dendritic synapses are inhibitory [45]. With the dendritic diameter estimated to be between 1 and 2 um, the dendritic surface area was in the order of 25000–50000 um2. Altogether, the dendrites and soma would be expected to receive roughly 600–1200 inhibitory synapses.

With a convergence of 600–1200 PCs, each firing at about 40 Hz, the DCN cells would be expected to receive PC-IPSP input at 24000–48000 Hz. A major effect is the reduction of the input resistance by one or two orders of magnitude, as compared to *in vitro* preparations. This, in turn, will for example affect the magnitude of the unitary IPSPs. Our data indicated that they were much too small to be resolved individually, probably well below 0.1 mV. This was indicated by the amplitudes of 15–20 mVs of maximal IO-evoked IPSPs (since these should correspond to the simultaneous activation of all PCs, a unitary PC IPSP would be in the range of 0.01–0.04 mV), the presence of IO-evoked IPSPs with peak amplitudes of 0.8 mV or smaller and the simulations of the membrane potential with spontaneous PC inputs.

The power spectral analysis showed that periods with low amplitude fluctuations are a natural consequence of the convergence on the DCN neuron of a large number of PC inhibitory synaptic inputs with low individual weights, which occurred with a low degree of synchronization. The resulting tonic inhibition can be counterbalanced by a cationic leak with a reversal potential of −30 mV, which is a dominating conductance in these neurons [46], and possibly also by background excitatory synaptic input [6].

### Concerted PC activation and the cerebellar microcomplex organization

It follows that the membrane potential of the DCN cell is in a highly dynamic equilibrium [4], [6], that could be rapidly changed as a result of any increase or decrease in PC activity. However, if each PC has a low influence, any substantial inhibitory or excitatory cortical modulation of a DCN cell requires a concerted increase or decrease in the activity of many of the PCs that innervate it. This is precisely what the microcomplex organization of the cerebellar olivo-cortico-nuclear circuitry provides for. In a cerebellar microcomplex, all PCs are innervated by climbing fibers activated from the same receptive field [19], [21]. The parallel fiber (PF) inputs to all the PCs of a microcomplex are normally from the same or closely functionally related peripheral sources, and also the inhibitory input to the PCs of a microcomplex is homogenous [39], [40], [47], presumably depending on cortical plasticity processes [38], [48], [49]. Thereby, the PCs of a microcomplex are likely to be activated and inhibited by similar behavioral events and their firing modulations will be concerted across the microcomplex. Behavioural studies have shown that this type of concerted PC activation among functionally related PCs does indeed occur [50].

### Relationship to previous *in vivo* studies of rebounds

In line with previous studies, we showed that IO stimulation can evoke rebound responses [14], [15]. However, with the cortical recordings of the climbing fiber and mossy fiber responses in PCs and granule cells, the present study provides a control of the extent of activated elements that was not provided in these previous studies. Presumably as the result of a more elaborated electrode placement, we could also obtain our maximal responses at much lower stimulation intensities (about one order of magnitude), which means more specific effects in terms of the elements stimulated. In our experiments, IO stimulation that evoked maximal DCN cell inhibition also evoked powerful rebound responses in the DCN cells. In contrast, submaximal IO-evoked inhibition consistently failed to evoke clear-cut rebounds (i.e. post-inhibitory excitation that exceeds 2 standard deviations of the pre-stimulus baseline). These findings are hence partly in line with the findings of [51], who questioned the role of rebounds *in vivo*, by confirming a relatively high threshold in terms of the (high) proportion of the afferent PCs that need to be activated with a high degree of synchrony to elicit the rebound response *in vivo*.

A recent study [14] could also confirm previous studies that inferior olivary stimulation [15] and high-intensity cortical stimulation [52] evoke rebound-like responses in the DCN. However, Hoebeek et al. used very high stimulation intensities and did not control the effects of the stimulation, raising the issue that the recorded responses may have been excitatory synaptic responses rather than a true rebound response. Train stimulation at current intensities of 150–300 uA is likely to activate more or less the entire brain stem of the mouse, in particular the pyramidal tract and the medial lemniscus. In the case of the cerebellar cortical stimulation [14], the intensity used would be expected to essentially activate the entire cerebellum. Both types of stimulations could set up substantial prolonged responses in the cerebral cortex, or other mossy fiber sources, which may spread to the cerebellum in the form of late excitation in DCN cells.

The presence of rebounds *in vivo* has been demonstrated directly by using hyperpolarizing current injections [14]. This issue was not systematically investigated here, but current induced hyperpolarization that would correspond to quite intense PC activation did not evoke rebounds (Fig. 1), in line with our other findings suggesting a high threshold for inducing rebounds in the adult cat *in vivo*. Furthermore, our rebound responses were not entirely congruent with the rebound responses from in vitro work, which has described one fast rebound response, attributed to T-type calcium channels [53], and a slow, prolonged rebound attributed to persistent sodium current [54]. Possible reasons for the discrepancy is that the channel composition in juvenile rodents, explored *in vitro*, and the adult cat *in vivo* could be different. Perhaps more importantly, the very different conductance state of the *in vivo* neuron could mean that the activation of these components is less distinct and may have different dynamics.

### Potential roles of the post-inhibitory excitation *in vivo*

We used manual peripheral activation of PF inputs to PCs but found that this type of input consistently failed to evoke rebounds in the DCN cells. Since the temporal response profile and depth of modulation of PC simple spike activation obtained with this stimulation (Fig. 8E and [40]) is not different from that recorded from PCs during different forms of behavioural modulation [29], [50], [55] this finding suggested that PF activation of PCs in the normal operational range may not be capable of eliciting rebounds. A hypothetical possibility is that the degree of simple spike synchrony between the different PCs of a microzone would be dramatically higher in the awake than in the decerebrate animal. However, speaking against this possibility is that the background PC simple spike activity in our preparation is very similar to that obtained in awake animals [29], [56], and the degree of coupling between climbing fibers is similar to that recorded in intact animals [35], [41], [57]. In addition, the findings obtained from IO stimulation in the present study suggest that rebounds require that a very large proportion of the PCs innervating the same DCN cell (i.e. PCs activated within the same sagittal band of one or a few microzones) are activated in near-perfect synchrony (i.e. within a couple of ms). Available data from awake animals show that simple spikes among pairs of PCs within the same sagittal, functional zone can be activated in relative synchrony during relevant behaviour but only with a coupling ratio of a few percent above chance [41].

In contrast, we found that synchronized activation of all the climbing fibers within the microcomplex evoked strong rebounds, which could suggest a role *in vivo* in association with climbing fiber activation. Climbing fiber responses can be readily recorded during motor performance [58], [59], [60], [61]. In addition, the climbing fiber system has natural mechanisms for synchronizing its activation, and climbing fibers within the same microcomplex display a heightened degree of synchrony [35], [62]. However, movement initiation is associated with a strong depression of IO excitability [58], [60], [61], [63] and during normal motor control, strong inhibition followed by rebound excitation is a pattern that is not observed in the DCN cell firing behaviour [59], [64]. A remaining possibility is that climbing fiber activation sufficiently synchronized to evoke rebounds occurs only when a strong afferent drive to the IO and synchronization of IO neurons coincide. This could for example be triggered by situations that require cerebellar learning. Alternatively, full-blown rebounds may be experimental artefacts in the sense that the degree of synchronous activation obtained by intracranial stimulation is unphysiological. The conductance machinery underlying the rebound could instead serve the purpose of speeding up the DCN cell response to situations that lead to a fast reduction in the PC simple spike output. This would help DCN cells to overcome the temporal limitations imposed by the lingering decay phases of the spontaneously activated PC IPSPs following inhibition from the cortical interneurons. Future studies are needed to resolve whether rebound responses actually occur under behavior or whether the conductance machinery that underlie them have other functions.

## Materials and Methods

Adult cats were prepared as previously described [40], [65]. Briefly, following an initial anesthesia with propofol (Diprivan® Zeneca Ltd, Macclesfield Cheshire, UK), the animals were decerebrated at the intercollicular level and the anesthesia was discontinued. The animals were artificially ventilated and the end-expiratory CO_2_, blood pressure and rectal temperature were continuously monitored and maintained within physiological limits. Mounting in a stereotaxic frame, drainage of cerebrospinal fluid, pneumothorax and clamping the spinal processes of a few cervical and lumbar vertebral bodies served to increase the mechanical stability of the preparation. Our EEG recordings were characterized by a background of periodic 1–4 Hz oscillatory activity, periodically interrupted by large-amplitude 7–14 Hz spindle oscillations lasting for 0.5 s or more. These forms of EEG activities are normally associated with deep stages of sleep. The pattern of EEG activity and the blood pressure remained stable, also on noxious stimulation, throughout experiments.

### Recordings and stimulation

The initial delineation of the forelimb area of the C3 zone in the cerebellar anterior lobe and the continuous monitoring of the general condition in the sensitive mossy fiber-to-granule cell-to-parallel fiber pathway were performed as described previously [39]. Also the general recording procedures and the procedures for placing stimulation electrodes in the inferior olive have been described in detail elsewhere [65]. In vivo patch clamp recordings were made from deep cerebellar nuclear cells with patch pipettes pulled to 6–14 MOhm (potassium-gluconate based internal solution, chloride 7.3 mM, same solution as in [65], [66]. A special adaptation for the DCN recordings was to pull the final 10 mm of the patch pipettes to one long, narrow part (<200 um) using a custom-made box-filament on a Sutter micropipette puller (P-97, Sutter Instruments Co., USA). Extracellular metal electrode recordings (exposed metal tips 3–15 um) were made from DCN neurons, Purkinje cells of the C3 zone, and climbing fiber field potentials in the molecular layer of the C3 zone.

In order to localize the anterior interposed nucleus (AIP), all experiments started with a topographical exploration using a metal electrode. The microelectrode was inserted at around the border between the C2 and C3 zones just rostral to the primary fissure at 90° angle relative to the horizontal stereotaxic plane. To keep track of the electrode location, we continuously monitored the spontaneous activity and the field and unitary responses evoked by electrical skin stimulation throughout electrode tracks. The dorsal border of the AIP was identified by a marked increase in background noise (presumably reflected neuronal multi-unit activity) relative to the overlying white matter, and the characteristic field potentials evoked by electrical skin stimulation [19], [67]. The medial and lateral borders of the AIP could be identified on the basis of the receptive field topography for climbing fiber-activated Purkinje cell inputs, as previously described [19], [67], [68]. The ventral part of the nucleus was characterized by a reduction in background noise.

Patch clamp pipettes were lowered under high positive pressure (3–10 atmospheres) until we reached the dorsal part of the nucleus according to the previous identification done with the metal electrode. Once inside the nucleus, the positive pressure was reduced but not removed. When dramatic increases in tip resistance occurred as the electrode was advanced, the positive pressure was removed and a seal formation was attempted. Once obtained (0.5–6 GOhm), rapid application of negative pressure was used to gain access to intracellular space (N = 24). The failure rate to both establish and break seals was markedly higher than in granule cells [66], [69]. In addition to the measures we have previously used, for DCN neuron recordings major quality checks for the recordings were the spike amplitudes (at least 35 mV) and the amplitudes of the spontaneous and IO-evoked giant IPSPs. Resting membrane potential was defined as the average potential recorded between spikes at 0 pA bias current within 30 s after intracellular access (−50 to −55 mV). Whenever spike amplitudes or giant IPSP amplitudes deteriorated by more than 20% from the initial record after we gained intracellular access, the recording was stopped since it was taken as an indication that the seal between the recording electrode and the cell membrane deteriorated. Access resistance was 7–15 MOhm and compensated off-line. Some neurons were stained by neurobiotin in the electrode solution. After sagittal sectioning, the neurons, which were always located in the AIP, were studied in the confocal microscope. The reconstruction shown in Fig. 1 was made from transparent z-stacks of confocal images from three contiguous sagittal sections. The full extent of the dendrites was not reconstructed, since the dendrites extended more than 0.4 mm in each direction perpendicular to the plane of section (sagittal), which made them very difficult to follow. Patch clamp recordings from granule cells and Purkinje cells were obtained using the same protocols as previously described [66], with the difference that Purkinje cells were recorded with electrodes with 4–8 MOhm resistance.

Input from the skin was quantified using pairs of closely spaced percutaneous needles (stimulation with one square pulse at 0.1 ms, 1 Hz, 1 mA) and a strain-gauge device mounted on the index finger of the investigator [65], [66].

Periods of inhibited spike activity and postinhibitory rebound spike responses were defined as coherent periods, that followed immediately after a spike inhibition (more than 2 standard deviations below prestimulus baseline), during which the spiking activity was increased relative to the prestimulus baseline activity by at least 2 standard deviations (SDs) for 10 ms or more.

In order to characterize the degree of randomness of the membrane potential over time, we used Welch's method to estimate the power spectral density (PSD) (Matlab). This analysis was applied to the intracellular activity in DCN cells prevented from firing using hyperpolarizing bias current and in the traces simulated by the model (see below).

We defined the onset of IPSPs (and EPSPs in granule cells) after computing the standard deviation of the second time derivative (smoothed by adjacent averaging of ±5 samples of digitized data) of the membrane potential during a 10 ms interval representing baseline membrane activity starting 5 ms before the onset of the PSP. The point when the recorded second time derivative first exceeded a value greater than 2 standard deviations over more than 5 sample bins (sample time 40 us) was taken as a probable start of a PSP. The potential PSP was then searched for a time-course typical for IPSPs (EPSPs in granule cells) as assessed by template-matching (using scalable EPSP templates created from isolated unitary EPSPs from the same recorded neuron using in-house software, see [69]) and if a match was found it was classified as an IPSP (/EPSP). The amplitude of a PSP was measured as the difference between the average membrane potential during a ±0.5 ms epoch around its the peak amplitude and the average membrane potential during a 1 ms epoch preceding the onset of the PSP.

Recordings of climbing fiber field potentials in the cerebellar cortex were made with a tungsten-in-glass microelectrode inserted in the superficial part of the cortex (inserted 100 um below the cortical surface). Each folium of the cortex was investigated with 15–20 recording spots within the C3 zone.

All data are given as mean ± standard deviation unless stated otherwise.

### Model of synaptic integration of IPSPs in DCN cells

A simple model of DCN cell synaptic integration, similar to that previously used for granule cells [66], [69], was used to calculate the summation of Purkinje cell IPSPs. The only purpose of this model was to analyze the pattern of membrane potential fluctuations which could be obtained as a result of varying the number and temporal density of Purkinje cell synaptic inputs. As the model was used for simulations within a very narrow membrane potential range, it was simplified to not include any active membrane conductances. Different Purkinje cell inputs were assigned one random out of 4 standardized IPSP amplitudes, with the largest IPSP having four times the amplitude of the smallest IPSP. The model assumed a background tonic excitation or leak to counterbalance the tonic inhibition level to bring the membrane potential to −50 mV on average (cf [4], [46]). The spike times of Purkinje cell simple spikes, recorded at a temporal resolution of 0.1 ms, was used to activate the IPSPs of the model. The simulation runs were made with 4, 13, 40, 120 and 600 PC inhibitory synapses, respectively.

### Ethics statement

The experimental procedures were approved in advance by the Malmö/Lund Animal Research Ethics Committee (permit number and approval-ID: M32-09).
